# Evaluation of Distress and Risk Perception Associated with COVID-19 in Vulnerable Groups

**DOI:** 10.3390/ijerph17249207

**Published:** 2020-12-09

**Authors:** Carmen Orte, Lidia Sánchez-Prieto, David Caldevilla Domínguez, Almudena Barrientos-Báez

**Affiliations:** 1 Department of Pedagogy and Specific Didactics, University of the Balearic Islands, Ctra. De Valldemossa km. 7.5. 07122 Palma de Mallorca, Spain; carmen.orte@uib.es; 2 Faculty of Information Science, Complutense University of Madrid, Avda. de Séneca, 28040 Madrid, Spain; davidcaldevilla@ccinf.ucm.es; 3 Iriarte University School of Tourism, University of La Laguna, 38400 Puerto de la Cruz, Spain; almudenabarrientos@iriarteuniversidad.es

**Keywords:** COVID-19, distress, risk perception, vulnerable groups, coping strategies, pandemic, preventive measures, information transmission, preventive behavior, BSI-18

## Abstract

Preventive behavior developed by the population is essential in the face of the risk of coronavirus infection (COVID-19). However, preventive measures will depend on the risk perception acquired. In addition, lockdown can directly affect mental health, provoking distress. Distress could affect risk perception. This study’s objective was to analyze whether experiencing distress had an influence on risk perception with respect to vulnerable groups. The sample consisted of 806 participants. The study was conducted during the first week of lockdown declared by the Spanish Government. The Brief Symptom Inventory BSI-18 and a risk perception questionnaire about vulnerable groups was administered. The study revealed the appearance of distress in 9.6% of the sample (85.7% women). Experiencing distress influenced risk perception. This study’s main contribution is the link between experiencing distress and the risk perception with respect to vulnerable groups. Risk perception is relevant since it can influence how the population faces the pandemic. Transmission of accurate information could help to minimize the effect of certain cognitive biases that affect risk perception and foster preventive behavior.

## 1. Introduction

### 1.1. Risk Perception during COVID-19

The preventive measures taken by the population are essential given the risk of Coronavirus infection (COVID-19) [1]. Specifically, the information received regarding risk and its perception around the epidemic will influence how it is faced [2], based on principles of health communication, such as those defined by Carreño-Salgado et al. (2011) [3] or Compte-Pujol et al. (2020) [4], and of communication during crisis moments studied by Hernández et al. (2019) [5] and Gómez (2011) [6], which are complicated given the impact of fake news [7]. Regarding pandemics, it is important to bear in mind the so-called optimism bias (absent in other similar cases, such as those studied by Aris, 2014 [8] or Ruíz et al., 2017 [9]) by which individuals estimate the probability of getting infected with the virus and infecting others if they do get infected. That is, this bias causes people to perceive themselves to be at lower risk of infection than someone similar to them [10,11,12]. This bias is also known as the “overconfidence effect”, and it was originally identified and substantiated by the authors Kahneman, Slovic, and Tversky (1982) [13]. The problem is that optimism bias interferes with the adequate execution of preventive measures (such as social distancing and hand washing), increasing the possibility of getting infected and infecting others [10,12].

According to the aforementioned, the data provided by the first studies addressing the population’s risk perception of COVID-19, conducted during 2020 (before March), indicated that the risk perception was low [11]. Most of the participants in a European survey opined that the risk was equal to or lower than 1% [12]. Regarding the risk perception surrounding COVID-19, to Jang et al. (2020) [14], affective risk perception, cognitive risk perception, and trust in the government had to be assessed, which are very different factors from those deemed relevant in other studies linked to distress, such as those of Rasti and Salajeghe (2019) [15] or Usheva and Filipova (2018) [16].

### 1.2. Vulnerable Groups

Bruine de Bruin (2020) established an association between internalizing symptoms (anxiety or depressive symptoms) and risk perception with respect to COVID-19 [17]. According to this author, risk perception would be a cognitive response to the experience of the pandemic, while the symptoms would be emotional responses [17]. Specifically, when highly stressful situations are experienced, as would be the case in a pandemic, distress has to be evaluated. Distress is a construct that assesses anxiety and depression symptoms, and somatization [18,19]. Therefore, both must be taken into account at the same time [14,20]. Additionally, a relationship between both responses can emerge; experiencing distress can also alter or modify risk perception [17].

Isolation conditions during the COVID-19 pandemic entailed significant changes in people’s everyday lives, having consequences not only for social aspects, but also for physical (physical safety) and psychological (mental well-being) ones [14,21]. Regarding mental health, anxiety, fear, depression, insomnia, and distress have been reported, as well as these being exacerbated where they already exist [1,14]. The study conducted by Wang et al. (2020) [22], demonstrated that, during the first phase of the outbreak in China, 53.8% rated the psychological impact as moderate to severe, 28.8% claimed having anxiety symptoms (moderate-severe), 16.5% experienced depressive symptoms (moderate-severe), and 8.1% suffered from stress (moderate-severe). The study conducted by Qiu et al. (2020) [18] reported that 35% of people surveyed experienced psychological distress since the COVID-19 pandemic initiated in China. The explanation for these percentages could be the anticipatory anxiety before the diagnosis of life-threatening diseases, which triggers unease, probably leading to distress [19]. Therefore, a high risk perception during a pandemic can also be negative, since it could lead to higher levels of distress [20].

In particular, risk perception and distress with respect to vulnerable groups must be taken into account. The main reason for this is that, as indicated by epidemiological studies, such as the one conducted by Chen et al. (2020) [23], there is a greater probability of the disease affecting older people or those with chronic diseases, since higher mortality rates are linked to: (a) old age, (b) obesity, and (c) comorbidity with other pathologies. That is, it is more likely for it to affect vulnerable groups. In fact, in the study carried out by Hu et al. (2020), which estimated COVID-19 transmissibility, found that risk of transmission was higher in adults and elder people [24]. In Spain, the number of deaths in vulnerable groups that occurred during the first wave of the pandemic (especially among the elderly) was particularly relevant. In fact, expert teams, such as the Novel Coronavirus Pneumonia Emergency Response Epidemiology Team, in March 2020, also sounded the alarm in the face of the number of infections and the consequences linked to the elderly [25].

Nevertheless, as stated in the study conducted by Chittleborough et al. (2011), it might be that this group could have a lower risk perception [20]. As demonstrated in the study by Bruine de Bruin (2020), which surveyed a sample made up of 6666 people, older people’s risk perceptions regarding COVID-19 were lower than those of other groups [17]. This could be linked to the Socioemotional Selectivity Theory, which states that older people are more predisposed towards maximizing their well-being due to their perception of having a shorter life time [26]. By the same token, the model of strength and vulnerability has also been identified, which posits that adult people, due to their trajectory and experiences, are more prepared to deal with difficulties and stressful events [27]. In any case, although it seems that the risk was higher for them during the first stage of the pandemic, their risk perceptions could be lower than those of other groups [20].

As has been explained before, the problem would be that risk perception could affect how the pandemic is faced, which could even affect the preventive measures taken [28]. In line with these reflections, the risk perceptions of these groups ought to be evaluated; however, in order to avoid “the overconfidence bias” or “the optimism bias” that might exist in these groups, such evaluation should be conducted based on third parties [10,11,12,13,14]. Deaux and Callaghan (1985), in their theoretical model, explain the importance of the evaluation being conducted based on key informants (third parties) to avoid influencing risk perceptions, especially, when addressing aspects related to health [29]. In the same way, it would be relevant to conduct the evaluation of distress, since it is a symptom that could affect directly risk perceptions [30]. Therefore, and as studied by Bruine de Bruin (2020) in the American population [17], the high infection rate in vulnerable groups forced evaluating whether it was associated with two variables: (a) with a low risk perception regarding infection (even adopting fewer protective measures), and (b) lower distress levels (which would directly affect risk perception). The reason lies in the fact that the emotional aspect (distress) of the vulnerable group could affect the cognitive aspect (risk perception) [17,31].

Therefore, with the aim of verifying the aforementioned hypothesis, three objectives were established: (a) to evaluate the percentage of people in the sample who suffered from distress during the first week of lockdown (during “pre-community outbreak phase”), (b) to identify whether there were significant differences between people who suffered distress and those who did not in their risk perception with respect to vulnerable groups, and (c) to identify whether age (belonging to a vulnerable group) influenced the risk perception with respect to vulnerable groups.

## 2. Methodology

### 2.1. Participants

A total of 806 people participated in this study. The sample mean age was 46.08 years old (SD = 15.31), with predominance of women (69.1%). The ages ranged from 25 to 59 years old (see Table 1). The age groups of the participants were established according to two variables: (a) being over 18 years of age, and (b) based on the mathematical model to quantify the age-specific transmissibility of the COVID-19 [24]. The mathematical model by Hu et al. (2020) found that transmissibility of the COVID-19 was higher among adults and elderly people (people over 60 years of age). Therefore, we considered as belonging to the vulnerable group based on age those people older than 60, as substantiated in the literature [24].

Most of the population surveyed was born in Spain (95.3%) and lived in the Balearic Islands (*n* = 583). A non-probabilistic snowball sampling was conducted, avoiding “the sampling bias” by establishing and selecting the first participants. As pointed out by Deaux and Callaghan (1985), we opted for key informants, who did not belong to the group under analysis, but that did know about the problem under evaluation. In this way, we avoided biasing the sample with the “optimism bias” previously mentioned [29].

Approximately half the sample did not have a significant other (being divorced, single, or widowed) (*n* = 408), but only 22.6% stated having no family (see Table 2). “Living with others” was predominantly with the spouse or significant other (37.6%). 25.2% of the sample were civil servants.

### 2.2. Design and Procedure

A cross-sectional type, quantitative methodology was used. The data were collected during the third week of March in Spain (from 17–23 March) coinciding with the first week of lockdown declared by the Spanish Government. Data collection was carried out on a digital platform, namely, through a questionnaire generating platform. The questionnaires were sent to participants’ mobile phones and e-mails, allowing them to respond it through any of both means.

Regarding the ethical protocol, the Ethics Committee of the Balearic Islands University examined and approved the study proposal. To participate in the survey, participants had to accept the terms and sign a consent form according to the Organic Law 3/2018, of 5 December, on the Protection of Personal Data and Guarantee of Digital Rights. They were informed about the use of their personal data, guaranteeing participants’ confidentiality.

Results were obtained by comparing the means, through the Student’s *t*-test. We also used ANOVA statistical test and a post-hoc test, using Tukey’s B test as a method for comparisons between groups.

### 2.3. Instruments

#### 2.3.1. Brief Symptom Inventory BSI-18

Brief Symptom Inventory 18 (BSI-18) (NCS Pearson, Minneapolis, EEUU) is a self-report that evaluates psychological distress and symptoms in both community and medical populations [32]. Specifically, the validation for the Spanish population was used [33]. It is an 18-item instrument, identifying the distress levels suffered in the last 7 days. The scores range from 0 to 72 points. Three main symptoms are evaluated: (a) anxiety, (b) depression, and (c) somatization. This instrument estimates the Global Severity Index (GSI), which determines whether a person suffers distress. Distress is considered a state of discomfort or anguish in which a person is unable to adapt to stressors being perceived or experienced.

BSI-18 has high reliability (α = 0.990). Internal consistency scores are adequate: for the somatization subscale, Cronbach’s α was α = 0.74, for the depression subscale α = 0.84, and for the anxiety subscale α = 0.79.

#### 2.3.2. Risk Perception Questionnaire Regarding Vulnerable Groups

A questionnaire evaluating the risk perception with respect to vulnerable groups was used. The development of an original questionnaire suitable for this study was necessary due to the COVID-19 being an emerging issue; hence, there were no empirically assessed or validated questionnaires available. The elaboration of the questionnaire based on two principal stages. The first stage was based on the review of literature focused on the subject [11,12,13,14,17,34,35]. In the second stage, a team of experts reviewed and adapted the questionnaire to the necessities of the Spanish culture, as well as its psychometric characteristics [36].

An exploratory factor analysis (principal components method) with Varimax orthogonal rotation was carried out based on Kaiser normalization. The KMO (Kaiser–Meyer–Olkin) test indicated a high relation between the correlation coefficients (KMO = 0.853) (see Table 3). Bartlett’s test of sphericity was significant, with a Chi-square value of 5512.331 (*p* < 0.001), thus indicating that the data are suited for factor analysis *(p* < 0.001).

The questionnaire is comprised of 17 items. The responses are evaluated using a Likert scale based on five types of answers: (1) strongly agree. (2) Agree. (3) Neither agree nor disagree. (4) Disagree. (5) Strongly disagree.

As a result of the factor analysis, four main factors were obtained: (a) Protective social-health and psychological measures, (b) Protection provided by competent authorities, (c) Higher risk level associated with vulnerable groups, and (d) Social awareness-raising about the protection of vulnerable groups. The four factors obtained from the factor analysis explained 58.89% of the total variance. Reliability analysis of factor 1 (built from 7 items) reached Cronbach’s α = 0.893. The reliabilities of the other factors were α = 0.722 for factor 2, α = 0.610 for factor 3, and α = 0.558 for factor 4 (see Table 4). Cronbach α values greater than 0.70 indicate a good internal consistency [37].

## 3. Results

### 3.1. Symptoms Experienced

The health alarm associated with the COVID-19 generated distress in 9.6% of the people (*n* = 77) in the sample analyzed. The mean age of the people who suffered distress was M = 38.06 (SD = 13.60). Significant differences between genders emerged (t (803) = −4.142; *p* < 0.001), with distress prevailing in women (85.7%). Experiencing distress was higher in people under the age of 60 (t (803) = 3.591; *p <* 0.001) (see Table 5).

### 3.2. Risk Perception with Respect to Vulnerable Groups

The participants stated that they “strongly agreed” with the fact that vulnerable groups must stay at home, that they are at higher risk, and that getting infected with the coronavirus can have repercussions for their health. This was especially noted in the following vulnerability indicators: (a) “vulnerable groups’ health will be severely damaged if they get infected with the coronavirus” (60.6% of participants), (b) “it is essential for vulnerable groups to stay at home (to be isolated)” (88.3% of participants), and (c) “vulnerable groups are at greater risk than other groups (those not considered high risk groups)” (64.7% of participants).

By the same token, a higher percentage of participants indicated that they “agreed” with citizens being aware of that vulnerability. This was identified according to the following indicators: (a) “there is social awareness of the importance of protecting vulnerable groups” (43.6% of participants), and (b) “citizens take into account the necessities of vulnerable groups” (41.7% of participants).

The people who experienced distress perceived a higher risk perception with respect to vulnerable groups than those participants that did not suffer distress. These results indicate that participants who experienced distress agreed to a lesser extent with the fact that the protective social-health and psychological measures for vulnerable groups were adequate (t (803) = −3.595; *p* < 0.001). They also stated that they agreed to a lesser extent with the fact that competent authorities protected vulnerable groups (t (803) = −2.452; *p* < 0.05). There were significant differences associated with the social awareness-raising factor; the people who did not experience distress stated that there was higher social awareness-raising than people who suffered distress (t (803) = −4.073; *p* < 0.001) (see Table 6).

Related to the risk perception with respect to vulnerable groups, there were differences according to participants’ age group. Regarding the protective social-health and psychological measures for vulnerable groups, the differences were significant between adults (between 25 and 59 years) and young people (between 15 and 24 years) (F (2, 802) = 3.731; *p* < 0.05). The young people group agreed to a lesser extent with the fact of adequate protective social-health and psychological measures being implemented for vulnerable groups (q = −0.205) compared to the adults group (q = −0.097) (see Table 7). Regarding the risk level around COVID-19, there were also significant differences between groups (F (2, 802) = 9.579; *p* < 0.001), with the young people group (G1) being the one that perceived lower risk associated with vulnerable groups (q = 0.364). On the other hand, older people (G3) perceived greater protection from competent authorities (q = −0.317) than the rest of participants, hence revealing significant differences (F (2, 802) = 11.689; *p* < 0.001).

## 4. Discussion

The study revealed that during the first week of lockdown in Spain (during the first phase of the pandemic), 9.6% of the sample was already suffering distress. Of those, 85.7% were women. Other studies, such as the one conducted by Liu et al. (2020) [21], also demonstrated that women had a tendency to suffer psychological distress in the face of the COVID-19.

However, distress did not prevail among people over 60 years of age (the sector of the population categorized as belonging to a high-risk group), but the highest distress percentage was linked to the adult population (ages from 25 to 59 years) (around 74%). The study conducted by Chittleborough et al. (2011) [20] also revealed a higher prevalence of psychological distress among younger people when patients had chronic conditions, placing greater importance on the age variable than on diseases. These results would fit the model of strength and vulnerability by Charles (2000), arguing that the accumulation of experiences would provide a person with the capacity to respond more effectively to adverse situations. This model highlights the importance of older people developing strategies to prevent symptoms and to promote emotional regulation when faced with negative situations [27]. However, there were also data contrary to the ones obtained in this study. For example, in the study conducted by Liu et al. (2020) [21], older people were the ones who experienced higher distress levels in the face of the COVID-19 pandemic. By the same token, the prevalence of distress during the SARS pandemic was also higher in older sectors of the population [38].

As stated above, experiencing distress influenced perceived vulnerability. Therefore, people who suffered distress perceived greater vulnerability in risk groups, particularly in association with three factors: (a) protective social-health and psychological measures, (b) protection provided by competent authorities, and (c) social awareness-raising. This conclusion coincides with Bruine de Bruin (2020) with respect to how distress could influence risk perception [17]. This aspect is highly relevant, because not only can suffering distress influence perceived vulnerability, but, as stated by Shigemura et al. (2020) [28], behaviors associated with fears or distorted risk perceptions might also influence emotional reactions, health risk behaviors, mental disorders and perceived health. In this sense, a negative thought loop could begin, by which distress would have an influence on perception and perception on distress. Therefore, risk perception is relevant since it can influence how people will face the epidemic [28]. Wang et al. (2020) [22] indicated that a strategy for preventing these symptoms could be providing to the population with accurate (focused on prevention strategies) and specific information about the region where the people live, while Forouzesh et al. (2019) [39] proposed individual strategies based on mindfulness, since it has consequences for psycho-social adjustment [40]. Similarly, it is recommended to maintain a daily routine, physical activity, and positive reappraisal/reframing [41] and avoid institutionalization [42].

It cannot be concluded that vulnerable groups were those who exhibited higher risk perception, since this could be due to the premises associated with “optimism bias” or “overconfidence bias” [10,11,12,13]. Therefore, even though the death toll and infection rates during the first stage of the pandemic in Spain indicated that vulnerable groups were at higher risk, and it was demonstrated in different studies [24,25] they did not show a higher risk perception; however, there were differences associated with the age groups when the assessment concerned third parties. Young people did state that vulnerable groups were exposed to a higher threat. These results are in line with the justification provided by Deaux and Callaghan (1985) [29], who highlight the importance of risk perception not being assessed solely by the vulnerable group, but also by key informants.

Finally, there were also significant differences in the age groups regarding the protective measures. While young people stated that the protective social-health and psychological measures were adequate, the group over 60 years of age (G3) was the one that was indicated to agree more with the fact that competent authorities provided protection to them. The acquisition of these preventive measures could be linked to greater trust placed in government/authorities, as verified in Seale et al. (2020) [43]. In fact, Qeadan et al. (2020) have already verified that the group of older people acquired more measures established by the competent authorities [44]. Therefore, authorities should consider the impact of their instructions and guidance, especially with respect to the group over 60 years of age.

## 5. Conclusions

This study’s main contribution is the link between the experience of psychological distress and risk perception with respect to vulnerable groups. It demonstrates that it would be relevant to intervene in the psychological distress being experienced, not only to reduce discomfort, but also to prevent risk perception from further entrenching itself. Distorted perceptions regarding vulnerable groups (which could include loved ones, such as relatives, friends, or even oneself) would increase the number of worries [31] or increase negative emotional reactions [41]. Promoting a realistic risk perception is essential, since it could affect how this pandemic is faced [45].

Psychological distress levels were moderate, probably because the evaluation was conducted during the first stage of the pandemic [18]. By 23 March 2020, the number of people infected was 35,316 in Spain, while currently (16 September 2020), 398,138 cases have already been registered. That is, the current status is that the number of people affected has soared. Therefore, if psychological distress and risk perception were evaluated today, they would probably exhibit worse figures. As mentioned previously, it is relevant to establish the date of data collection since it is linked to higher levels of optimism or pessimism in the face of the epidemic. As stated by Lau et al. (2010) [46], during the “pre-community outbreak phase” of the pandemic caused by SARS, they registered that only 6% of the Korean population experienced psychological distress. However, it would be advisable to measure the levels experienced during the spike of the pandemic or when it ends. In fact, Peng et al. (2010) [38] found higher levels of pessimism 6 months after resolution of the SARS pandemic in Taiwan. Finally, this study was conducted, specifically with a population from the Balearic Islands; hence, in order to make these conclusions more generalizable to the Spanish population, a more representative sample of the Spanish people should be collected. Consequently, as future lines of research, we propose conducting a new evaluation (post-test) that incorporates possible variations produced in the risk perception according to the new conditions experienced in Spain.

Therefore, as pointed out by Goulia (2010) [31], it will be necessary to take into account the experience of psychological distress and risk perception, in both current and future pandemics. The acquisition of a more realistic perception that allows the acquisition of appropriate coping strategies based on the prevention of infection most be promoted. In addition, those perceptions that lead to apprehension, distress or discomfort, based on distortions of reality or information overload, must be prevented.

## Figures and Tables

**Table 1:** Characteristics of the sample.

Characteristics		*n*	%
Gender	Man	248	30.8
	Woman	556	69.1
	Other	1	0.1
Age groups	18- to-24-year-olds	84	10.4
	25- to-59-year-olds	545	67.7
	Older than 60	176	21.9
Birth country	Spain	767	95.3
	Outside Spain	38	4.7
Autonomous Community where the person lives	Balearic Islands	583	72.4
	Other Spanish Autonomous Communities	15	1.9

**Table 2:** Social, educational and occupational characteristics of the population.

Characteristics		*n*	%
Civil Status	Single	287	35.7
	Married or with significant other	398	49.4
	Divorced	103	12.8
	Widowed	17	2.1
Family composition	With no family	182	22.6
	Spouse or significant other	177	22
	Spouse or significant other and children	346	43
	Children	81	10.1
	Other relatives	19	2.3
Living with others	Alone	119	14.8
	Parents	91	11.3
	Spouse or significant other	303	37.6
	Spouse or significant other and children	50	6.2
	Children	127	15.8
	Other relatives	104	12.9
	Non-relatives	10	1.2
	Residence	1	0.1
Job	Non-specialized laborer	5	0.6
	Specialized worker or laborer	34	4.2
	Middle management or administrative	69	8.6
	Manager or director	31	3.9
	Employed professional	119	14.8
	Self-employed o autonomous professional	62	7.7
	Landlord	10	1.2
	Civil servant	203	25.2
	Researcher	4	0.5
	Student	30	3.7
	With no job	105	13
	Other	133	16.5

**Table 3:** Kaiser–Meyer–Olkin Test and y Bartlett’s Test of Sphericity.

Exploratory Factor Analysis		
Kaiser–Meyer–Olkin Measure of Sampling Adequacy		0.853
Bartlett’s Test of Sphericity	Approx. Chi-Square	5512.331
	Df	136
	Sig.	0.001

**Table 4:** Correlations obtained from rotated component matrix of the factor analysis.

Vulnerability Questionnaire Items about Vulnerable Groups	Components			
	1	2	3	4
Factor 1: Protective social-health and psychological measures				
The current social measures in my Community fulfill the necessities of vulnerable groups.	**0.855**		0.072	
The current health measures in my Community fulfill the necessities of vulnerable groups.	**0.841**		0.058	0.108
The current social measures in my Community can fulfill the necessities of vulnerable groups	**0.816**	0.179		0.077
The current health measures in my community can fulfill the necessities of vulnerable groups	**0.787**	0.218		0.090
I think vulnerable groups receive emotional support from the community in the current situation	**0.612**	0.457		
The information provided about the protective measures that vulnerable groups need are sufficient	**0.600**	0.329		0.163
Vulnerable groups can access the food, hygiene, or self-care resources they require.	**0.563**	0.482	−0.111	0.110
Factor 2. Protection provided by competent authorities				
Vulnerable groups have protective measures at their disposal	0.295	**0.731**		
Competent authorities have taken measures to protect vulnerable groups	0.473	**0.549**		0.129
Factor 3. Higher risk level associated with vulnerable groups				
Vulnerable groups are at greater risk than other groups that are not considered high risk	0.052	−0.142	**0.694**	0.222
Vulnerable groups’ health will be severely damaged if they get infected with the virus	0.055	−0.147	**0.685**	0.121
Risk of infection for vulnerable groups is high in my Community	−0.109	0.296	**0.605**	0.091
Risk of getting worse at a hospital is high for vulnerable groups	−0.164	0.393	**0.604**	−0.105
I think the measures implemented must be more dedicated to the necessities of vulnerable groups	0.262	−0.181	**0.508**	−0.222
Factor 4. Social awareness-raising about the protection of vulnerable groups.				
It is essential for vulnerable groups to stay at home (to be isolated)		−0.143	0.240	**0.677**
There is social awareness of the importance of protecting vulnerable groups	0.408	0.334		**0.595**
Citizens take into account the necessities of vulnerable groups	0.378	0.459	−0.051	**0.573**

**Table 5:** Characteristics of the sample who suffered distress.

	Do Not Suffer Distress		Suffer Distress				95% Confidence Interval on the Difference	
	M	SD	M	SD	t (803)	*p*	Lower Bound	Upper Bound
Gender	1.200	0.400	1.077	0.269	−4.142	0.001	−0.268	−0.094
Older than 60 years of age	1.675	0.471	1.857	0.352	3.591	0.001	0.054	0.190

**Table 6:** Risk perception based on whether there was distress.

	Do Not Suffer Distress		Suffer Distress				95% Confidence Interval on the Difference	
	M	SD	M	SD	t (803)	*p*	Lower Bound	Upper Bound
Protective social-health and psychological measures	−0.040	0.085	0.386	1.05	−3.595	0.001	−0.661	−0.194
Protection provided by competent authorities	−0.028	1.00	0.264	0.058	−2.452	0.014	−0.527	−0.058
Vulnerability level associated with vulnerable groups	0.004	0.995	−0.044	1.044	0.415	0.678	−0.185	0.285
Social awareness-raising about the protection of vulnerable groups	−0.046	0.967	0.437	1.188	−4.073	0.001	−0.716	−0.250

**Table 7:** Risk perception according to age group.

	15-to-24-Year-Olds		25-to-59-Year-Olds		Older Than 60 Years of Age				
	M	SD	M	SD	M	SD	F (2, 802)	*p*	Tukey Test
Protective social-health and psychological measures	−0.205	0.943	0.063	1.020	−0.097	0.071	3.731	0.024	G1; G2 < G2; G3
Protection provided by competent authorities	0.104	1.022	0.086	0.976	−0.317	1.003	11.689	0.001	G3 < G2; G1
Higher risk level associated with vulnerable groups	0.364	1.194	−0.095	0.943	0.121	1.02	9.579	0.001	G2; G3 < G1
Social awareness-raising about the protection of vulnerable groups	0.160	0.980	−0.026	0.972	0.004	1.08	1.280	0.279	G1; G2; G3
